# Oral L-citrulline supplementation enhances cycling time trial performance in healthy well-trained males

**DOI:** 10.1186/1550-2783-12-S1-P52

**Published:** 2015-09-21

**Authors:** Takashi Suzuki, Masahiko Morita, Yoshinori Kobayashi, Ayako Kamimura

**Affiliations:** 1 Healthcare Products, Development Center, KYOWA HAKKO BIO Co., Ltd, Tokyo, Japan; 2 Laboratory of Pharmacognosy, School of Pharmaceutical Sciences, Kitasato University, Tokyo, Japan

## Background

L-citrulline is an amino acid that is an endogenous precursor of L-arginine and contributes to generating nitric oxide (NO). L-citrulline is known for increasing plasma L-arginine and NO more effectively than equivalent doses of L-arginine. NO plays an important role in sport performance but it is presently unknown whether L-citrulline enhances sport performance during rowing ergometer competition in humans. The aim of this study is to investigate the effect of oral supplementation of L-citrulline on cycling time trial performance.

## Methods

A randomized double-blind crossover study design was used. Twenty two well-trained males, aged between 20 and 39, consumed 2.4 g / day of L-citrulline or placebo for 7 days and they took 2.4 g of L-citrulline or placebo 1 hour before 4 km cycling time trial on day 8. Completion time of 4 km cycling, power output / VO_2 _ratio (PO / VO_2_), plasma NOx, amino acids, Visual Analog Scale (VAS) were evaluated.

## Results

L-citrulline supplementation significantly improved cycling time trial performance by 1.5% (p < 0.05) and increased PO / VO_2 _during performance (p < 0.1). Moreover there was a correlation between plasma NOx and PO / VO_2 _(r = 0.47, p < 0.01) in L-citrulline group. L-citrulline significantly increased plasma L-citrulline and L-arginine, and improved the subjective feeling of muscular fatigue and concentration (p < 0.05).

## Conclusion

Oral L-citrulline supplementation enhances cycling time trial performance by improving PO / VO_2 _through up-regulation of plasma NO availability.

